# Skin Diseases and Syphilis

**Published:** 1893-11

**Authors:** 


### *SELECTIONS AND ABSTRACTS.*

### SKIN DISEASES AND SYPHILIS.

EDITED BY DR. M. B. HUTCHINS.

### THE CLINICAL HISTORY OF A CASE OF XERODERMA PIGMENTOSUM.

E. C., aged (when first seen, May 4, 1892) thirty-four years eight months; lawyer; in robust general health but reduced about thirty pounds as the result of numerous caustic applications by a "cancer doctor." No history of other skin disease or illness. He had fair complexion, blue eyes, hair of a lighter shade than the beard—both getting gray (the latter thin). The hair afterwards grew rapidly gray. Height, six feet two and a half inches, weight 180 pounds, normal weight, 210 pounds. The only living child of first cousins. He had a sister who died of hemorrhagic malarial fever at fourteen. Uncle of his mother and father died of "cancer." Mother's father had a "chronic sore" on the face, but died of malaria and kidney disease. Mother living, in fairly good health. His father died of pneumonia at thirty-five (patient then aged six). He sometimes drank to excess. This is also said to be true of the patient. Was reared on a farm but did not work—went to school.

*Primary Lesions.*—The disease began on the face at eighteen, as well as he can remember, as "little, rough, scaly spots" averaging the size of a flake of bran, gradually enlarging peripherally. Removal of the scales exposed a thin, gummy exudation. He said the majority of the lesions persisted in this form. When examined—over both cheeks, especially above horizontal lines from the oral angles to the ears, slightly in bearded portion of the face, on the forehead, scattering on the sides and back of the neck, the following lesions: Dark brown, pigmentary spots, from half pea to dime in diameter, the smaller as simple lentigines, enlarging they become depressed and show a network of fine vessels—telangiectasis. Proceeding further, some of them become adherently crusted with dry, sebaceous matter, and may become reddened, with a more or less distinct areola, slight ulceration occurring in points beneath the crusts. These lesions were as large as a silver quarter. The new growths are supposed to have originated from this form. Their crusts varied in color from dark brown to almost black. Where the lesions are most numerous there is a general, fine telangiectasis of the skin. Intermingled with the above lesions are a few simple apigmentary points, with or without telangiectasis, or slight atrophy. Another form of lesion, which he said was primary, is that of pea to dime diameter, charactized by simple, slight, papillary hypertrophy, the papules about the size of a small millet seed, the lesions well defined, covered with a thin, adherent scale or crust, the removal of which without wounding the papillæ was difficult. Some of these were free of scale in their early stage. In some of the atrophic, apigmentary spots minute, anemic papillæ are seen through their thin epidermic covering. The sebaceous follicles on each side of the nose, in the naso-labial furrows and beneath the lower lip plugged with a blackish, dry, sebaceous substance. Patches of plates of a similar substance on each side of the bridge of the nose; present for the past two weeks. On the back of the left hand a number of large, lentigo-like lesions, and one dry, slightly scaly, papular point. On the back of the right hand a number of roundish, atrophic, apigmentary lesions, a few slightly scaly points and a few of the usual pigmentary lesions. No telangiectasis on either hand. The disease is limited at the wrists, then there are a few lentigo-like points on the arms. The hands are as shapely, soft, smooth and hairless as a woman's. The skin beneath the clothing very fair, soft and smooth. The latter part of June it was noted that the lesions appeared less marked, and some, as the fine papular on the forehead, had either atrophied or simply subsided. The blackish crusting on the nose disappeared in a few weeks under the use of a (carbolic?) salve by the patient. Later, when the patient was confined entirely to the house, all the above described lesions appeared still fainter, save one or two of the older, crusted ones; but when seen a month later there was an increase of the sebaceous crusting on the older lesions, and a tendency to faint papulation in some of the intermediate ones. This tendency to oscillation between better and worse was characteristic throughout. The older lesions showed no vestige of pigmentation, save in the crusts. I saw him for the last time in October, just three months before his death. The older lesions showed a persistence of the dry, sebaceous crusting, while many of the apparently more recent were scarcely visible. During the time he was under observation there did not appear to be any development of new lesions of the kind described. No local treatment was employed for the "skin trouble," my efforts having been directed to the management of the "new growths." Had any "new growths" appeared in the primary lesions, it was my intention to attempt their entire removal by excision. The actual course of the lesions was not always well remembered by the patient or easy of definement by myself. The characteristic localization to the exposed parts of the skin, the parts which usually "freckle," is worthy of note.

*The New Growths.*—The first of these appeared twelve years before, at twenty-two, four years after first noticing the other lesions, over the middle of the border of the body of the lower jaw—left side—as a pea-sized, warty growth which gradually enlarged during eight years until it reached the diameter of a silver quarter. It had then become a raised "swelling," the surface open, irregular, discharging a mucoid, offensive substance which dried into thin, loose, temporary crusts. A "cancer doctor" then destroyed it with a "vegetable paste." It recurred in twelve months at the posterior superior margin of the scar, possessing the same characteristics as before. Another "cancer doctor" removed this with an unknown "paste." Recurrence in six months. Treated by the same man—apparently more successfully. A livid, furrow-like scar following the border of the jaw marked the seat of the diseased growth. This scar was two inches long and half an inch wide, and showed a close network of dilated vessels. At the posterior end of this just above the angle of the jaw, was a linear depression, reddened, not a part of the scar, its sides showing a number of small sebaceous crusts; and a pea diameter, cicatricial point at the upper end. A cutaneous horn on the left side of the nose was destroyed by a "paste" five or six years before, leaving a marked, smooth, white scar. About four years before a half pea-sized growth developed beneath the right ear over the anterior border of the sterno-cleido-mastoid muscle, soon became ulcerated and depressed and discharged a yellowish-red, mucilaginous, fetid substance. At about the same time a somewhat similar growth appeared over the ramus of the right jaw. This was destroyed by a paste in April, 1888. The one beneath the ear was first treated ("paste") a year before, and not having been cured, sixteen similar applications were made from December, 1891, to April, 1892, during which torture he became addicted to the morphine habit. When these notes were made there remained, at the site of the "burnings," an unhealthy opening the size of the finger-tip, surrounded by a livid, brawny infilteration. The tragus of the right ear became diseased and was excised in 1884. Growing from this site, when examined, was a globular, livid, elastic "tumor," sensitive to the touch, filling the cavity of the concha; a small opening below from which oozed a thin, yellowish, odorless fluid. This growth had developed in the preceding three weeks. In front of this, below the right zygoma, a diffuse, livid, rather elastic swelling, seated upon which were a number of dark, thin, loose crusts. The scar of the first tragus operation separated this and the present tragal growth. In 1884 the actual cautery was also used in this region, but with bad effect. About the same time a depressed, crusted lesion was excised just below the right malar bone, leaving a deep, smooth scar.

May 4, 1892, I removed the tragus growth—under ether—antiseptic precautions, using the knife and scissors to cut through its origin. This was composed of firm tissue; the remainder, the globular portion, consisted of a thin, cutaneous capsule enclosing a soft, brain-like substance easily removable, down to the bottom of the conchal cavity, with the curette. The external auditory meatus was found involved. Iodoform and iodoform gauze used as a dressing. Five days later a little pus was noticed, and at tragal margin a beginning growth. At the end of three weeks the wound was "about healed," but the recurrent growth was the size of a large pea, having its seat in the skin. This was removed, under cocaine, a week later. Beneath the skin covering was the same soft, granular material as in the former growth. Removed all with the curette and thoroughly swabbed the wound with pure carbolic acid. Twelve days later the wound showed unhealthy, easily bleeding granulations. A week after this a growth had begun to form in the antitragus, and the operated wound and all about the meatus were unhealthy, granular, oozing sero-pus, and the tissues below, including the numerously burned place, were livid, infiltrated, brawny. A few waxy, shot-sized papules appeared on the posterior surface of the concha. The latter never changed during my observation of the case.

The opening below the ear was cauterized several times with the nitrate of silver stick, and kept packed with iodoform gauze. It never healed—filled with exuberant granulations; these broke down: pus formed, and so on in rotation, finally reaching in depth almost to the cervical column. As the diseased conditions of the tissues increased and involved the region of articulation of the jaw (as well as the right side of the face), the patient was unable to separate the jaws more than half an inch. A scratch in the scar on the right cheek was followed by the formation of pus and crusting. The point above the left maxillary angle became papular, ulcerated, crusted. Progressive weakness and the more or less constant pain in the right aural region kept the patient in bed most of the time after the first two months. The antitragus growth gradually involved the lobe of the ear, while the entire region around and including the meatus was in an unhealthy granular, papular, ulcerative condition, and the tragal region was undermined. Once (only) a cheesy substance was pressed out of the sub-aural opening. The livid, brawny condition described persisted—better or worse—throughout, until it broke down. The destructive process gradually advanced in all directions, and upon one occasion a marked "carcinomatous" odor was observed. When I visited him at his home in a distant town, in August, the right facial nerve had become involved, producing complete paralysis of the muscles it supplied, the right half of the lip was swollen, and there was an almost constant flow of saliva from this side.

When last seen, October, 1892, the destruction had reached the depth of the bony meatus, to the middle of the zygoma in front, and most of the lobe of the right ear was destroyed through breaking down of the last new growth, and the mastoid process was almost exposed.

The patient died of exhaustion three months later, fearfully emaciated. There was no hemorrhage from any of the vessels destroyed by the disease. His family physician wrote that, at the end, the surface destroyed extended from the outer canthus of the right eye down, including the ear, nearly to the clavicle.

*Microscopic Examination.*—Unfortunately, every specimen prepared from the two growths removed was lost in changing my office, and I have to trust to my memory of examinations made last summer. Most of the sections were stained with borax-carmine, some from the last tumor with osmic acid and mounted in glycerine, without having been in alcohol (were frozen for section). No malignant disease was apparent in any of the sections. There was simply an atheromatous condition, with an apparent hypertrophy and numerical increase of the sebaceous glands. The sebaceous glands were stained a dark brown in the osmic preparations, and the lower layers of the rete almost as brown. The brain-like substance was composed of granular cells, staining poorly, and detritus, as of partly broken down sebaceous glands and contents. It was impossible to get other specimens or a *post-mortem.*

*Treatment* is worthy of mention only to demonstrate further two already well known facts, viz.: its futility in this disease, and the impossibility of getting it followed by patients in many. Syrup of iodide of iron was taken for a time. "Asiatic pills" were badly borne and he abandoned them. Fowler's solution "disagreed," and he stopped taking it. He continued his morphine, taking as much as a small coffee spoonful at a dose, one to three times in twenty-four hours. During the last few months the wound was dressed twice a day with a twenty per cent. solution of ichthyol (stronger than ordered, but continued when it was found not to irritate). His physician was accustomed to add a little morphine to one dressing each day. Pure carbolic acid was occasionally applied to the sub-aural cavity.

His mother had a few faint, brownish spots about the orbits and forehead, and one over the right zygoma covered with dry, adherent scales. These lesions bore considerable resemblance to those on her son's face, but also resembled those often seen on the face of old persons.

Every element of the patient's disease has been described, and as the clinical history speaks for itself, it is scarcely necessary to call attention to any special feature. The age at which the trouble appeared to have begun and the time of appearance of the "new growths," as well as the age which the patient attained, with the disease, are remarkable. The want of aptness of any particular title for the disease is shown by the numerous names which we find given it by different authorities. I leave it to some master of descriptive titles to suggest a thoroughly suitable name for this class of cases.—Dr. M. B. Hutchins, in *Journal of Cutaneous and Genito-Urinary Diseases, October, 1893.*

